# Direct Synthesis of Single‐Crystalline LiNi_0.8_Co_0.1_Mn_0.1_O_2_ Cathodes From Lithium‐Containing Precursors via Spray Pyrolysis

**DOI:** 10.1002/smsc.70397

**Published:** 2026-09-03

**Authors:** Tae Ha Kim, Yeon Oh Lee, Yeong Beom Kim, Hyungsub Kim, Jung Sang Cho, Sang Mun Jeong, Yun Chan Kang, Gi Dae Park

**Affiliations:** ^1^ Department of Urban, Energy and Environmental Engineering Chungbuk National University Cheongju Chungbuk Republic of Korea; ^2^ Department of Advanced Materials Engineering Chungbuk National University Cheongju Chungbuk Republic of Korea; ^3^ Department of Materials Science and Engineering Korea University Seongbuk‐Gu Seoul Republic of Korea; ^4^ Neutron Science Division Korea Atomic Energy Research Institute (KAERI) Daejeon Republic of Korea; ^5^ Advanced Energy Research Institute Chungbuk National University Cheongju Chungbuk Republic of Korea; ^6^ Department of Engineering Chemistry Chungbuk National University Cheongju Chungbuk Republic of Korea; ^7^ Department of Chemical Engineering Chungbuk National University Cheongju Chungbuk Republic of Korea

**Keywords:** Li‐containing precursor, Li‐ion batteries, Ni‐rich layered cathode, single‐crystalline cathode, spray pyrolysis

## Abstract

Single‐crystalline cathode materials have attracted considerable attention for addressing the cycling instability of Ni‐rich layered cathode materials caused by anisotropic lattice strain during repeated cycling. However, conventional synthesis methods for single‐crystalline cathodes generally require high temperatures and prolonged calcination times. Alternative approaches, such as multistep lithiation, molten‐salt, and hydrothermal methods, still suffer from limitations such as complex processes, mandatory washing, and challenges in large‐scale production. Therefore, in this study, a direct synthesis strategy for single‐crystalline LiNi_0.8_Co_0.1_Mn_0.1_O_2_ (SC‐NCM) cathode materials was proposed using Li‐containing precursors prepared by spray pyrolysis. Because lithium was homogeneously incorporated within the precursor particles, SC‐NCM was successfully synthesized through direct calcination without additional Li‐mixing or washing processes. As a result, single‐crystalline NCM was successfully crystallized at 900 °C for 3 h with 700 °C for 6 h healing sequence and exhibited superior cycling performance compared with polycrystalline NCM (PC‐NCM), delivering a capacity retention of 81% after 1000 cycles at 0.5 C. Therefore, this work provides an effective approach to simultaneously address both the intrinsic limitations of Ni‐rich layered cathode materials and the challenges associated with conventional synthesis processes.

## Graphical abstract

A novel strategy for the direct synthesis of single‐crystalline LiNi_0.8_Co_0.1_Mn_0.1_O_2_ cathodes via spray pyrolysis is introduced, delivering stable cycling performance. Spray pyrolysis enables the synthesis of Li‐containing precursors and allows short‐duration calcination without additional Li mixing. The homogeneous elemental distribution and formation of single‐crystal particles lead to outstanding cycling stability in lithium‐ion batteries.

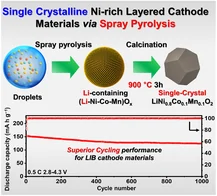

## 1 Introduction

Ni‐rich layered cathode materials have attracted significant attention for lithium‐ion batteries used in energy storage systems and electric vehicles due to their high energy density [1, 2, 3]. However, Ni‐rich layered materials undergo anisotropic expansion and contraction along the c‐axis direction during repeated cycling, resulting in severe structural degradation such as microcracking at grain boundaries, transition metal dissolution, oxygen release, and eventual pulverization of secondary particles [4, 5, 6, 7, 8]. Due to these intrinsic limitations, conventional polycrystalline Ni‐rich layered cathode materials suffer from loss of electrical contact, hindered Li^+^ diffusion pathways, and rapid capacity fading [9, 10, 11]. To address these limitations, single‐crystalline Ni‐rich layered cathode materials have been proposed. Unlike polycrystalline materials composed of aggregated primary particles, single‐crystalline cathodes consist of discrete single‐crystal particles, which effectively suppress microcrack formation and particle pulverization, thereby enhancing structural stability and cycling performance [12, 13].

Conventionally, the synthesis of single‐crystal cathode materials requires high calcination temperatures and prolonged heat treatment times to induce sufficient grain growth [13, 14]. However, such conditions inevitably increase process costs and can accelerate structural degradation in Ni‐rich layered materials. In particular, Ni‐rich cathodes are highly susceptible to cation mixing and rock salt‐like phase transitions during prolonged high‐temperature treatment [15, 16]. To overcome these challenges, various synthesis methods, including multistep lithiation, molten‐salt, and hydrothermal methods, have been reported [17, 18, 19, 20, 21, 22]. However, multistep lithiation methods require complex synthesis procedures involving repeated calcination, additional Li‐mixing, and annealing processes. Molten‐salt methods have attracted attention because of their relatively simple synthesis procedures and reduced processing temperatures. However, residual flux salts, such as LiCl, NaCl, and KCl, must be removed through additional washing processes after calcination. In addition, hydrothermal methods generally suffer from limitations in large‐scale production and require additional separation processes between liquid and solid phases.

To overcome these limitations, spray pyrolysis has attracted considerable attention for the synthesis of cathode materials because of its homogeneous elemental distribution, scalability for large‐scale production, and minimal wastewater generation [13, 23, 24, 25, 26]. However, maintaining a stable particle morphology during calcination remains challenging because severe necking and particle coalescence frequently occur during crystallization. Consequently, previous studies have predominantly reported polycrystalline cathode materials synthesized by spray pyrolysis rather than single‐crystalline cathodes [27, 28, 29, 30, 31]. Therefore, in this study, we introduce a synthesis strategy for single‐crystalline Ni‐rich layered cathode materials using Li‐containing precursors prepared by spray pyrolysis, followed by short‐duration calcination. Unlike conventional coprecipitation methods that require an additional Li‐mixing process, Li‐, Ni‐, Co‐, and Mn‐containing oxide precursors synthesized by spray pyrolysis inherently contain uniformly distributed lithium within each particle, enabling the direct synthesis of layered cathode materials through calcination. Because lithium is homogeneously incorporated throughout the precursor particles, Li^+^ diffusion occurs over substantially shortened diffusion distances during calcination compared with conventional solid‐state lithiation processes. Consequently, grain growth and crystallization can be achieved within a significantly reduced heat treatment time, enabling the formation of single‐crystalline layered cathode materials. Through the proposed synthesis strategy, both the intrinsic limitations of Ni‐rich layered cathode materials and the drawbacks of conventional synthesis methods, such as prolonged processing times, additional Li‐mixing procedures, and complex synthesis routes, can be effectively addressed. Compared with polycrystalline NCM synthesized by spray pyrolysis under relatively low‐temperature calcination conditions, the proposed single‐crystalline NCM exhibits significantly enhanced cycling stability. In this study, a spray pyrolysis‐based Li‐containing precursor strategy is proposed for the synthesis of single‐crystalline Ni‐rich layered cathode materials through direct calcination.

## 2 Results

**SCHEME 1:**
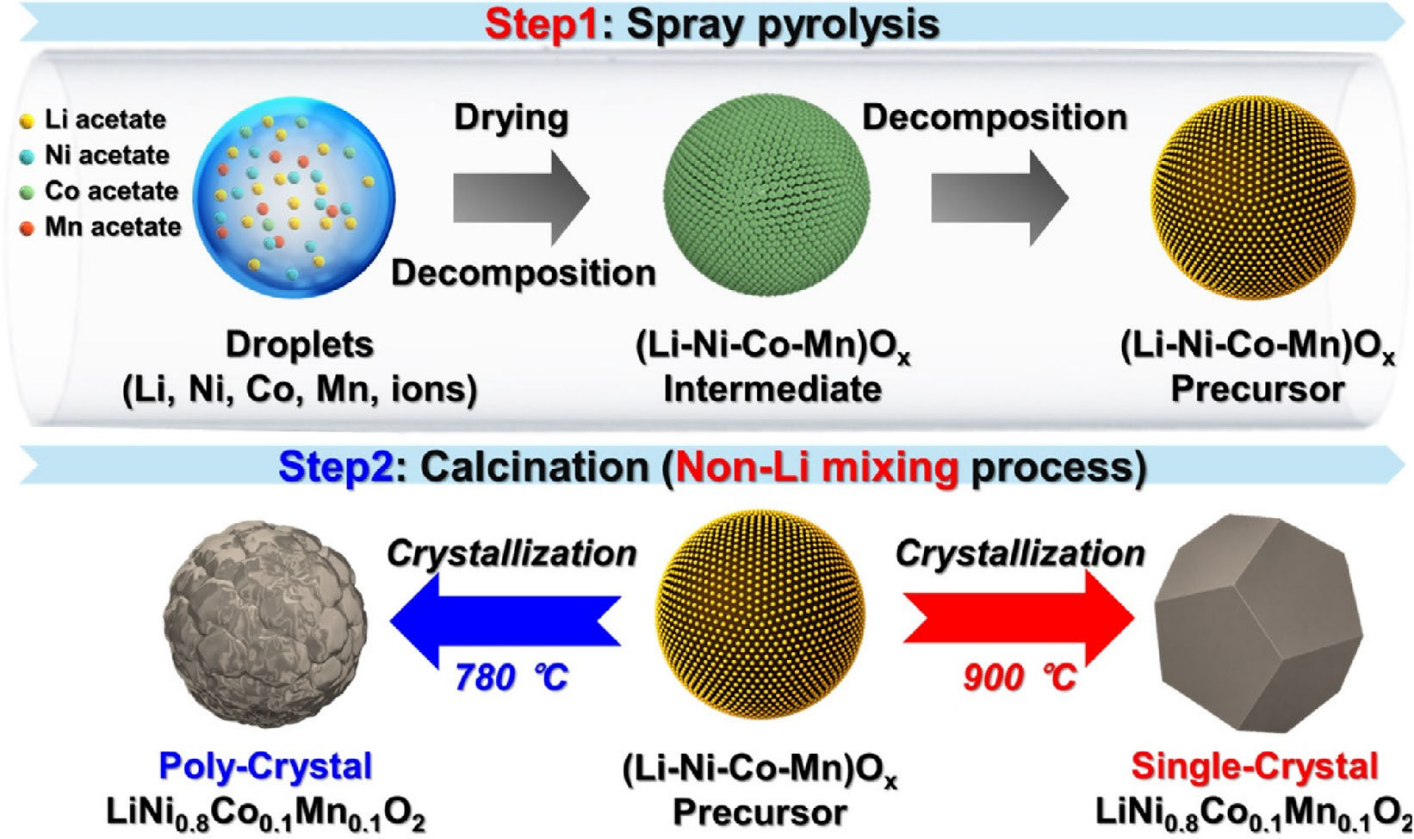
Schematic illustration of the formation mechanism using spray pyrolysis and calcination processes for PC‐NCM and SC‐NCM.

A spray pyrolysis‐based synthesis strategy for single‐crystalline Ni‐rich layered cathode materials using Li‐containing precursors and high‐temperature calcination is shown in Scheme 1. Li, Ni, Co, and Mn acetate salts are completely ionized in distilled water to form a homogeneous precursor solution. The rationale for employing spray pyrolysis to prepare the Li‐containing precursor is that the droplets generated by the ultrasonic nebulizer act as micro‐reactors that promote a uniform distribution of all elements. During spray pyrolysis, Li, Ni, Co, and Mn are present in an ionized state within the aqueous droplets, and each droplet serves as an individual micro‐reactor in which the thermal reaction and diffusion of all elements occur homogeneously. Consequently, Li‐containing spherical precursors are successfully obtained through the one‐pot spray pyrolysis process. Because lithium is already incorporated within the precursor particles, the as‐prepared precursors can be directly calcined to synthesize Ni‐rich layered cathode materials without an additional Li‐mixing process, unlike the conventional coprecipitation method. Furthermore, the homogeneous distribution of Li and transition metal elements within the precursor particles effectively shortens the Li diffusion pathway during calcination, thereby facilitating grain growth and rapid crystallization of layered structures for the formation of single‐crystal cathode materials. Depending on the calcination conditions, different crystallization behaviors are observed for single‐crystalline and polycrystalline structures. Under high‐temperature conditions (900 °C for 3 h), sufficient thermal energy is provided for grain growth within individual particles, resulting in the formation of single‐crystal NCM materials. In contrast, relatively low‐temperature calcination conditions (780 °C for 15 h) do not provide sufficient driving force for large grain growth into single‐crystal particles. Instead, polycrystalline secondary particles are formed through severe necking and aggregation between primary particles during prolonged calcination. Compared with conventional coprecipitation methods and previously reported synthesis strategies, the Li‐containing precursor approach provides favorable conditions for single‐crystal formation through reduced Li diffusion pathways during calcination.

SEM (Scanning electron microscopy) images and XRD (X‐ray diffraction) patterns of precursors are shown in Figure S1. Due to the droplet‐derived characteristics of spray pyrolysis, the precursors exhibit spherical morphologies, as shown in Figure S1a,b. In Figure S1c, the XRD patterns of the NCM precursors display broad NiO peaks without any detectable impurity phases, indicating that NiO‐based precursors are successfully synthesized through spray pyrolysis at 300 °C. The spray pyrolysis temperature was set as low as 300 °C since a highly crystalline precursor hinders the transformation of the cubic NiO phase into the layered structure, while an excessively high temperature promotes Li vaporization from the Li‐containing precursor. Owing to the poor crystallinity of the precursor prepared by spray pyrolysis at 300 °C, Li‐related reflections are rarely detected in the XRD patterns. Instead, to demonstrate the presence and distribution of Li in the precursor, X‐ray photoelectron spectroscopy (XPS) analysis of the Li 1s region was conducted on the NCM precursor, as shown in Figure S2. Because the Li 1s region overlaps in binding energy with the transition metal 3p levels, the spectra were deconvoluted into Mn 3p (~48 eV), LiOH (~55 eV), Co 3p (~60 eV), and Ni 3p (~67 eV) contributions [32, 33, 34, 35]. The LiOH component confirms the presence of Li in the precursor particles.

Before the calcination process for transforming the precursors into layered cathode materials, thermogravimetric analysis (TGA) was conducted to investigate the thermal decomposition and reaction behavior of the precursors, as shown in Figure S1d. As reported in previous studies, Li surface reactions, melting and diffusion, topotactic lithiation, and layered structure transformation are critical processes for obtaining highly ordered layered structures with minimized cation mixing and oxygen vacancies [36, 37, 38, 39]. Based on the TGA results, intermediate heat treatment temperatures of 200, 250, 300, and 500 °C were selected to induce Li surface reactions, Li melting, and lithiation reactions while suppressing the formation of Li_2_O and other by‐products. Finally, subsequent calcination processes at 780 and 900 °C for 15 and 3 h, respectively, were conducted to form polycrystalline and single‐crystal layered cathode materials (denoted as PC‐NCM and SC‐NCM), respectively. Furthermore, Ni‐rich layered cathodes suffer from cation mixing and defect formation during the high‐temperature calcination required for single‐crystal synthesis [13, 15]. To address this issue, a relatively low‐temperature calcination conducted after the high‐temperature calcination can restore these defects [12, 40, 41]. Indeed, the sample calcined at 900 °C for 3 h without the subsequent 700 °C 6 h step exhibited a lower *I*_(003)_/*I*_(104)_ ratio than that of SC‐NCM that underwent the 700 °C 6 h healing process (Figure S3).

**FIGURE 1:**
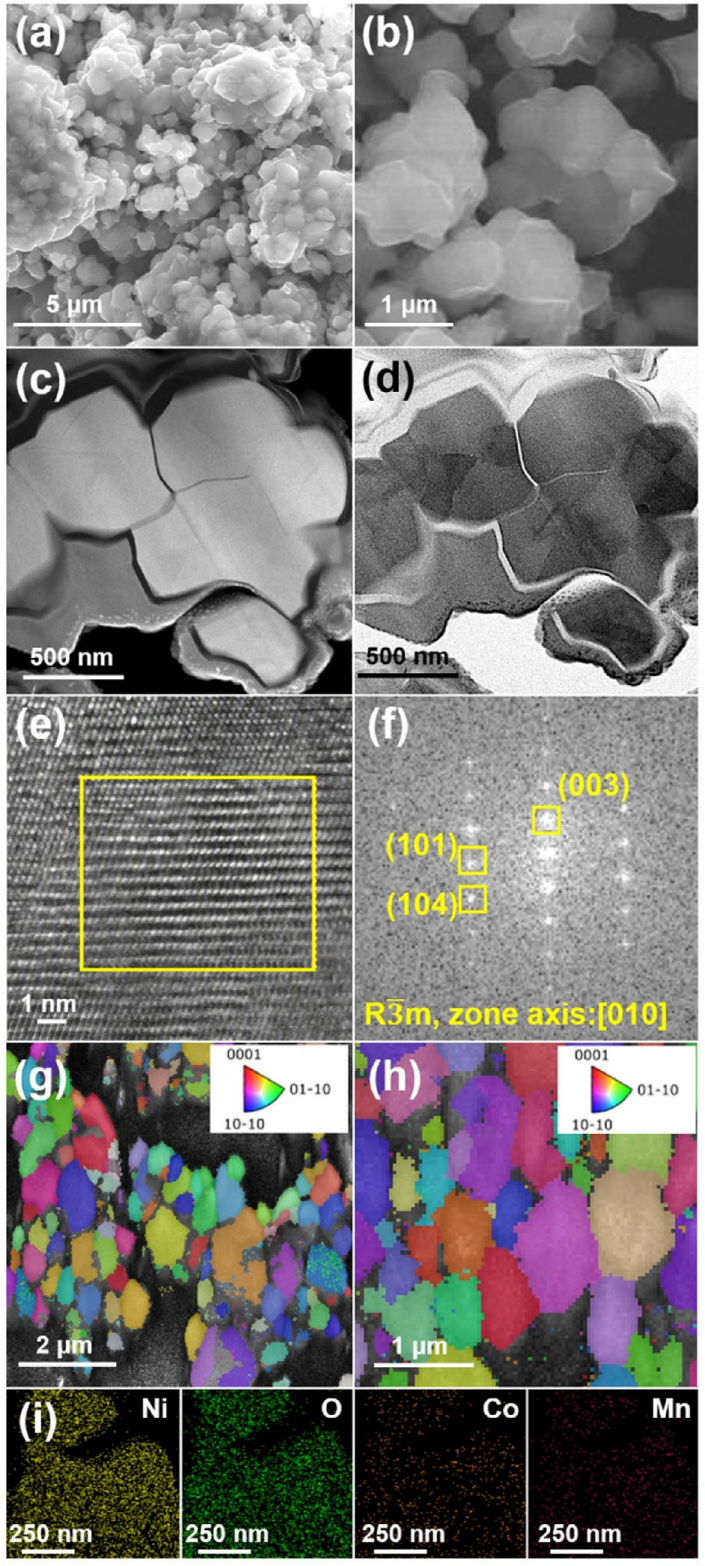
Morphological properties and crystal structure of PC‐NCM: (a,b) SEM images, (c) dark‐field STEM image, (d) bright‐field STEM image, (e) HR‐TEM image, (f) corresponding FFT pattern, (g,h) EBSD IPF maps, and (i) elemental mapping images.

In Figure 1, morphological properties and crystal structures of PC‐NCM are displayed. SEM images of PC‐NCM exhibit agglomerated polycrystalline microspheres, as shown in Figure 1a,b. Because relatively low‐temperature long‐duration calcination conditions (780 °C for 15 h) do not provide sufficient driving force for complete grain growth into single‐crystal particles, crystallization and severe necking occur between adjacent precursor particles during calcination. STEM (Scanning transmission electron microscopy) bright‐field and dark‐field images obtained from FIB (focused ion beam)‐prepared cross sections are shown in Figure 1c,d. These images clearly reveal inner grain boundaries within individual particles and interconnected primary particles, confirming the polycrystalline materials. HR‐TEM (high‐resolution transmission electron microscopy) and FFT (fast Fourier transform) analyses obtained from the selected area (yellow box) further verify the formation of a layered structure, corresponding to the (003), (101), and (104) planes indexed with the [010] zone axis, as shown in Figure 1e,f. Furthermore, to investigate the detailed grain distribution within the particles, EBSD (electron backscatter diffraction) inverse pole figure (IPF) maps reveal multiple grains with different crystallographic orientations within individual particles. The presence of multiple grain boundaries within particles further confirms the polycrystalline PC‐NCM, and it is consistent with the SEM and STEM observations. Energy‐dispersive X‐ray spectroscopy (EDS) elemental mapping images show uniformly distributed Ni, Co, Mn, and O elements throughout the particles, indicating that a homogeneous elemental distribution was successfully achieved during the spray pyrolysis process.

**FIGURE 2:**
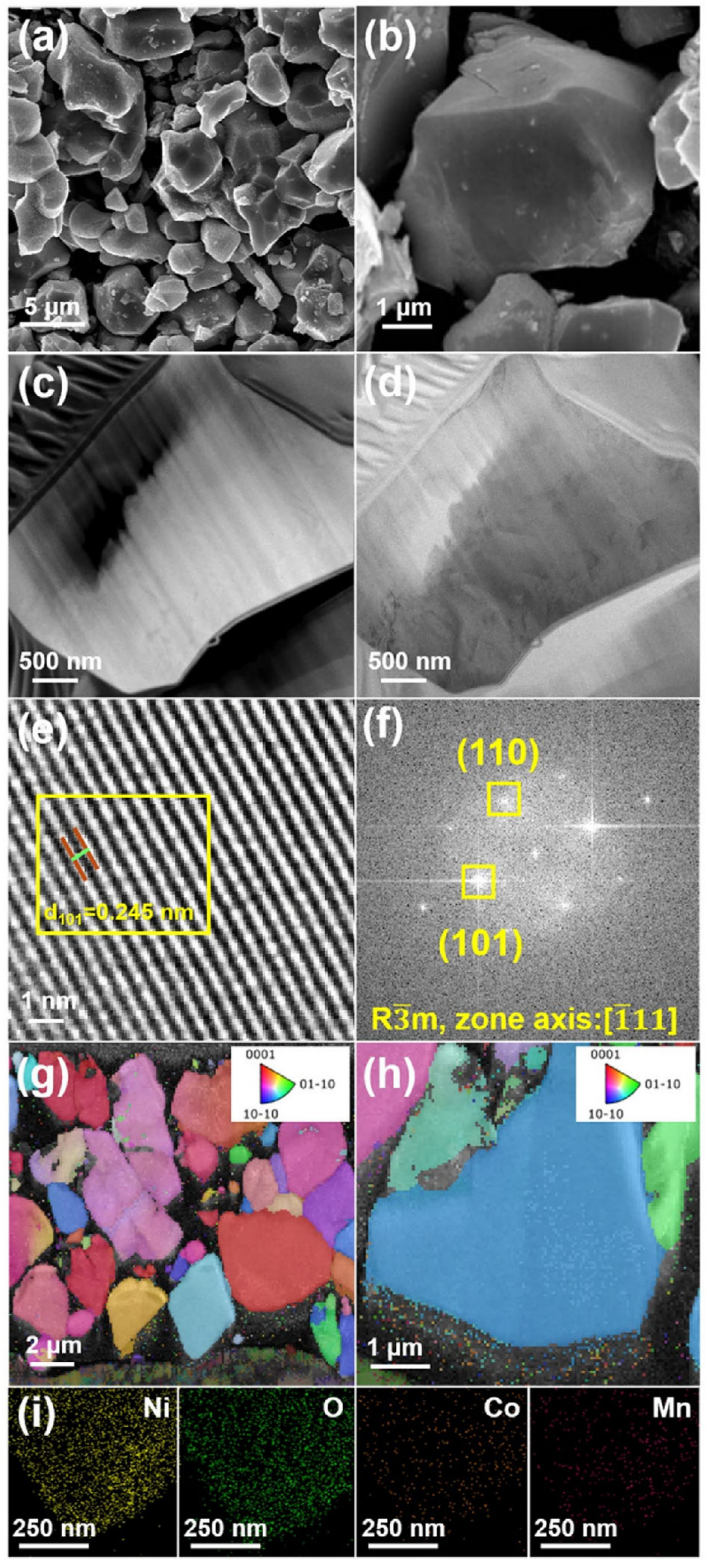
Morphological properties and crystal structure of SC‐NCM: (a,b) SEM images, (c) dark‐field STEM image, (d) bright‐field STEM image, (e) HR‐TEM image, (f) corresponding FFT pattern, (g,h) EBSD IPF maps, and (i) elemental mapping images.

Unlike PC‐NCM, SEM images of SC‐NCM exhibit isolated single‐particle morphologies, as shown in Figure 2a,b. In contrast to the interconnected morphologies observed in PC‐NCM, SC‐NCM consists of separated large particles with sizes of approximately 3 μm without noticeable particle agglomeration. The high‐temperature, short‐duration calcination condition (900 °C for 3 h) provides sufficient thermal energy for grain growth within individual particles, resulting in the formation of single‐crystal NCM particles. Furthermore, STEM bright‐field and dark‐field images shown in Figure 2c,d clearly confirm the large single‐grain structure of SC‐NCM. HR‐TEM analysis obtained from the selected area (yellow box) further verifies the formation of a layered structure, showing a clear (101) lattice plane with a d‐spacing of 0.245 nm, as shown in Figure 2e. The corresponding FFT pattern displayed the (101) and (110) planes indexed with the [$\bar{1}11$] zone axis, as shown in Figure 2f. The EBSD IPF results exhibit isolated crystallographic orientation throughout each particle without distinct internal grain boundaries, confirming the successful formation of single‐crystal NCM particles. In addition, EDS elemental mapping images of SC‐NCM show uniformly distributed Ni, Co, Mn, and O elements throughout the particles, indicating that a homogeneous elemental distribution is maintained even after high‐temperature calcination.

The inductively coupled plasma optical emission spectroscopy (ICP‐OES) data shown in Table S1 indicate that Li loss during spray pyrolysis is negligible, resulting in a precursor composition close to the designed stoichiometry. The precursor exhibited Li/Ni/Co/Mn ratios of 1.12/80.803/10.062/9.135. After calcination, PC‐NCM and SC‐NCM maintained an appropriate stoichiometry with compositions of 1.020/79.728/10.216/10.056 and 1.006/79.034/10.208/10.757, respectively. These results indicate that the spray pyrolysis process effectively preserves the designed elemental composition while enabling the formation of both polycrystalline and single‐crystal layered NCM materials.

XRD patterns and Rietveld refinement results of PC‐NCM and SC‐NCM are shown in Figures S3a, S4, and 3a,b. In XRD patterns, both samples exhibit well‐developed crystallinity corresponding to the layered α‐NaFeO_2_ phase structure without detectable impurity phases in the hexagonal R3̅m space group. Notably, unlike the conventional Li solid‐state mixing process, which requires additional washing and calcination steps, both SC‐NCM and PC‐NCM prepared from the spray pyrolysis precursors showed no Li impurity phases in their XRD patterns even without any washing process, supporting the uniform distribution of Li within the particles.

**FIGURE 3:**
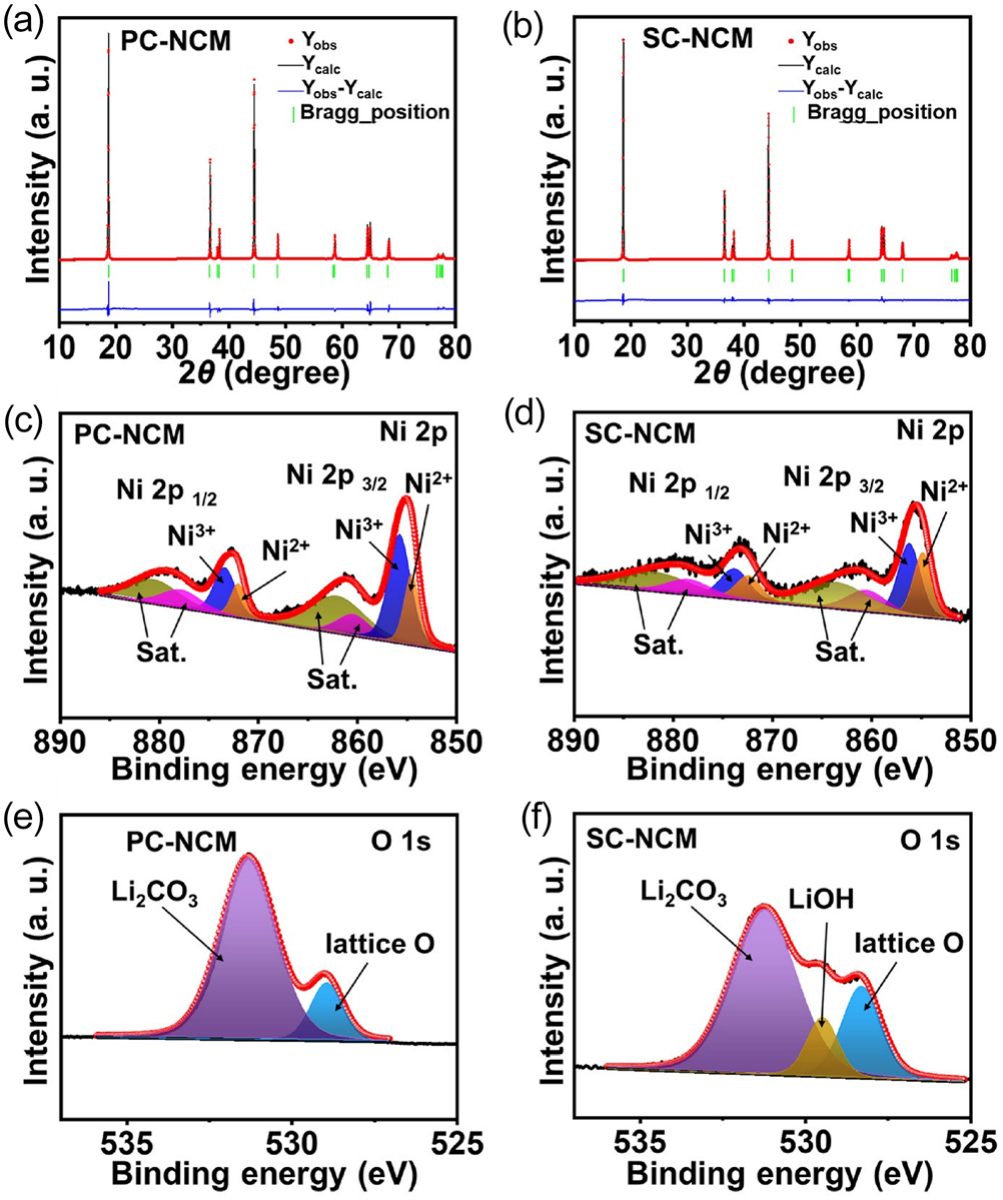
Crystal structure and chemical states of PC‐NCM and SC‐NCM: (a,b) Rietveld refinement and (c–f) XPS spectra corresponding to (c,d) Ni 2p and (e,f) O 1s.

Detailed Rietveld refinement results, including lattice parameters, cell volume, and cation mixing degree, are shown in Table S2. The lattice parameters of the a‐axis and c‐axis are calculated to be 2.86977 and 14.1949 Å for PC‐NCM and 2.87986 and 14.2198 Å for SC‐NCM, respectively. Due to sufficient grain growth under high‐temperature calcination conditions, SC‐NCM exhibits expanded lattice parameters and a larger unit cell volume of 102.133 Å^3^ compared with 101.241 Å^3^ for PC‐NCM. Generally, prolonged high‐temperature calcination of Ni‐rich layered cathode materials induces severe cation mixing because of Li evaporation and increased Ni^2+^ formation. Accordingly, SC‐NCM exhibits a relatively higher cation mixing degree of 2.8% compared with 1.0% for PC‐NCM.

To investigate the detailed chemical states of both samples, XPS analyses were conducted, as shown in Figure 3c,d. The Ni 2p spectra of both samples reveal characteristic Ni 2p_1/2_ and Ni 2p_3/2_ corresponding to the Ni^2+^ and Ni^3+^ states with satellite peaks (denoted as Sat.). PC‐NCM exhibited a higher Ni^3+^ percentage than Ni^2+^ (69.2% and 30.8%, respectively), with Ni^2+^ peaks located at 854.5 and 872.03 eV and Ni^3+^ peaks located at 855.76 and 873.4 eV [42, 43]. In contrast, SC‐NCM exhibited a relatively lower Ni^3+^ percentage than PC‐NCM, with Ni^3+^ and Ni^2+^ fractions of 56.8% and 43.2%, respectively. The Ni^2+^ peaks of SC‐NCM were located at 854.83 and 872.4 eV, while the Ni^3+^ peaks were located at 856.22 and 873.83 eV. The increased Ni^2+^ fraction in SC‐NCM indicates that high‐temperature calcination promoted partial Ni^2+^ formation during crystallization, which is consistent with the relatively higher cation mixing degree obtained from the Rietveld refinement results. Nevertheless, both cathode materials maintain relatively low Ni^2+^ ratios, indicating that severe cation mixing is effectively suppressed despite different calcination conditions. In the O 1s spectra, both PC‐NCM and SC‐NCM exhibit characteristic peaks corresponding to Li_2_CO_3_ species formed by surface Li reactions with air atmosphere and lattice oxygen. The fitted peaks of PC‐NCM located at 531.32 and 528.95 eV are attributed to Li_2_CO_3_ and lattice oxygen, respectively [44]. Similarly, SC‐NCM also exhibits Li_2_CO_3_ and lattice oxygen peaks located at 531.25 and 528.31 eV, respectively, together with a relatively weak LiOH‐related peak at 529.49 eV [44, 45, 46]. This difference in lithium species originates not from the amount of residual lithium but from the degree of its conversion with CO_2_. Upon air exposure, surface residual lithium reacts with moisture to form LiOH, which is subsequently converted into the more stable Li_2_CO_3_ through further reaction with CO_2_ [47, 48]. Because PC‐NCM consists of smaller primary particles with a higher grain boundary density, it provides a larger reactive surface area, so that its lithium is more extensively converted to Li_2_CO_3_. In contrast, SC‐NCM, which possesses a considerably lower grain boundary density, undergoes less conversion and thus retains a larger fraction of the intermediate LiOH.

**FIGURE 4:**
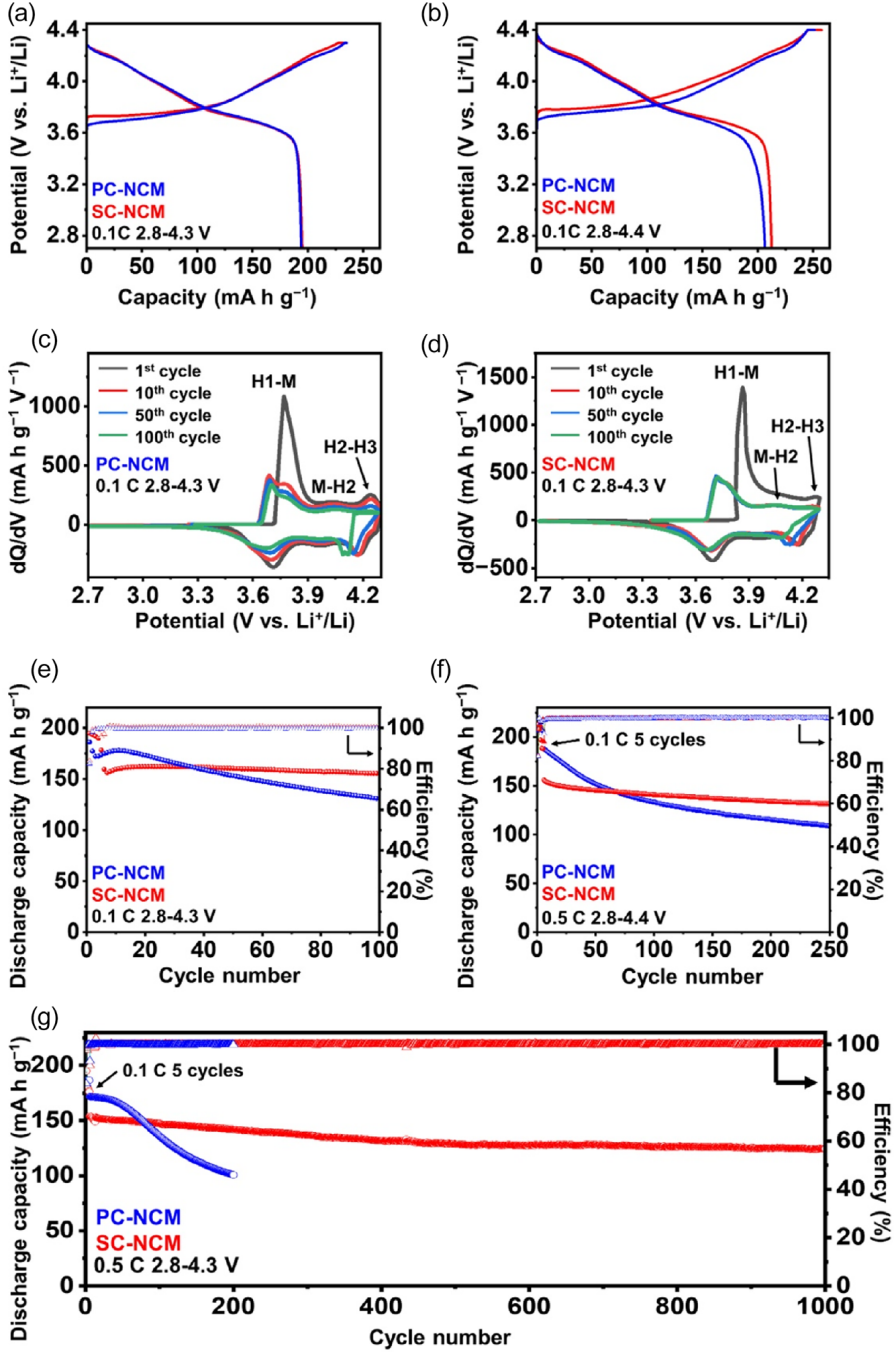
Electrochemical performances of PC‐NCM and SC‐NCM: (a,b) initial charge–discharge curves; (c,d) dQ/dV curves; cycling performances measured with (e) 4.3 V cutoff voltage at 0.1 C, (f) 4.4 V cutoff voltage at 0.5 C, and (g) 4.3 V cutoff voltage at 0.5 C.

Electrochemical performances of PC‐NCM and SC‐NCM are shown in Figure 4. Galvanostatic charge–discharge tests, galvanostatic differential capacity (dQ/dV) analyses, and cycling tests were conducted. Initial charge–discharge curves of both samples exhibit analogous tendencies under both 4.3 and 4.4 V cutoff, as shown in Figure 4a,b. The initial charge and discharge capacities of PC‐NCM at 2.7–4.3 and 4.4 V were 235.08/194.15 and 251.35/206.61 mA h g^−1^, corresponding to ICEs (initial Coulombic efficiencies) of 83% and 82%, respectively. In the case of SC‐NCM, the initial charge and discharge capacities were 232.38/194.95 and 257.77/212.61 mA h g^−1^ under the same voltage windows, corresponding to ICE of 84% and 82%, respectively. Under the 4.3 V cutoff condition, PC‐NCM and SC‐NCM exhibited similar initial discharge capacity. However, under the 4.4 V cutoff condition, SC‐NCM displayed a higher initial discharge capacity compared with PC‐NCM. These results imply that the single‐crystal particle structure of SC‐NCM provides enhanced resistance against anisotropic lattice strain during high‐voltage charge processes.

To investigate the electrochemical reaction mechanism during cycling, dQ/dV curves of PC‐NCM and SC‐NCM measured at the 1st, 10th, 50th, and 100th cycles are shown in Figure 4c,d. Both samples exhibit characteristic phase transition‐related peaks reported in previous studies [49, 50]. Distinct peaks observed between 3.7 and 4.3 V correspond to the H1–M–H2–H3 phase transitions during the charge process [50, 51]. Notably, the distinct H2–H3 phase transition peak near 4.3 V gradually decreases during repeated cycling, which is associated with poor reversibility of the H2–H3 phase transition and progressive structural degradation [52, 53]. However, SC‐NCM exhibits relatively broadened H2–H3 phase transition peaks during both the initial and repeated cycles compared with PC‐NCM. This broader peak separation is caused by the sluggish bulk diffusion in single‐crystalline cathodes and the relatively higher degree of cation mixing in SC‐NCM rather than by an intrinsic loss of redox reversibility. Nevertheless, the lower initial polarization of PC‐NCM does not guarantee cycling stability, since the repetitive anisotropic lattice strain accumulated in PC‐NCM progressively generates intergranular microcracks, which provide penetration pathways for electrolyte infiltration and continuously increase the interfacial resistance. Because PC‐NCM is more vulnerable to anisotropic stress and particle cracking, distinct H2–H3 phase transition peaks, peak intensity reduction, and peak position shifts are more significantly observed during cycling. Likewise, cyclic voltammetry (CV) curves measured within 2.7–4.3 V at a scan rate of 0.1 mV s^−1^ also exhibit analogous phase transition‐related peaks, as shown in Figure S5. In particular, the distinct H2–H3 phase transition peak near 4.2 V observed in PC‐NCM is significantly suppressed in SC‐NCM. These results indicate that single‐crystal particles effectively suppress crack formation induced by lattice volume variation and internal stress accumulation along grain boundaries, thereby mitigating unfavorable H2–H3 phase transitions during repeated cycling [54]. As a result of the cycling tests, SC‐NCM exhibited significantly improved cycling stability compared with PC‐NCM under various current densities and voltage cutoff conditions. In Figure 4e, under the 4.3 V cutoff condition at 0.1 C, PC‐NCM initially delivered a slightly higher discharge capacity due to its relatively large surface area and sluggish bulk Li diffusion kinetics of SC‐NCM [55]. However, continuous cycling rapidly deteriorated the capacity retention of PC‐NCM, resulting in lower discharge capacities than SC‐NCM after approximately 40 cycles. Finally, the capacity retentions of PC‐NCM and SC‐NCM were 76% and 80%, respectively. Likewise, under the 4.4 V cutoff condition at 0.5 C, PC‐NCM exhibited much more severe capacity fading, as shown in Figure 4f. PC‐NCM and SC‐NCM exhibited distinct differences in capacity retention of 58% and 84%, respectively, after 250 cycles. Notably, SC‐NCM maintained excellent long‐term cycling stability up to 1000 cycles at 0.5 C, sustaining a capacity retention of 81%, whereas PC‐NCM retained only 59% after 200 cycles. Furthermore, to investigate high‐voltage stability, additional cycling tests of SC‐NCM were conducted under a 4.5 V cutoff condition at 0.5 C, as shown in Figure S6. SC‐NCM still exhibited initial charge and discharge capacities of 256.83/208.17 mA h g^−1^, corresponding to an ICE of 81% and stable cycling performance up to 150 cycles with a capacity retention of 74%. In contrast, PC‐NCM delivered initial charge and discharge capacities of 275.45/225.07 mA h g^−1^, corresponding to an ICE of 82%, and exhibited a capacity retention of 67% after 150 cycles. These results demonstrate that SC‐NCM maintains more stable cycling performance under the 4.5 V cutoff condition, since its single‐crystalline nature effectively suppresses the anisotropic strain generated during high‐voltage charging.

The advantage of the Li‐containing precursor was verified using a Li‐free precursor, which was prepared via spray pyrolysis, mechanically mixed with a lithium salt, and calcined under the same conditions as those used for SC‐NCM. As shown in Figure S7, the SEM images of the Li‐free precursors exhibited dense spherical morphologies. After calcination, the resulting sample exhibited nonuniform single‐crystal formation, in which single‐crystalline and polycrystalline particles were observed together in the SEM images. This indicates that the solid‐state mixing hinders uniform single‐crystal formation. Furthermore, the XRD pattern of the solid‐state Li‐mixed sample exhibited Li‐related impurity peaks such as those of Li_2_O and Li_2_CO_3_. As results of the electrochemical performance, the solid‐state Li‐mixed sample delivered low initial charge and discharge capacities at 0.1 C in the voltage range from 2.7 to 4.3 V. Owing to the electrochemically inactive residual Li impurities, it delivered low charge and discharge capacities of 177.77 and 150.19 mA h g^−1^, respectively, corresponding to an ICE of 84%. In addition, it retained a low discharge capacity of 80.49 mA h g^−1^ at 0.5 C after 75 cycles. Therefore, the Li‐containing precursor prepared via spray pyrolysis facilitates the formation of single‐crystalline cathodes without the formation of Li‐related impurities.

**FIGURE 5:**
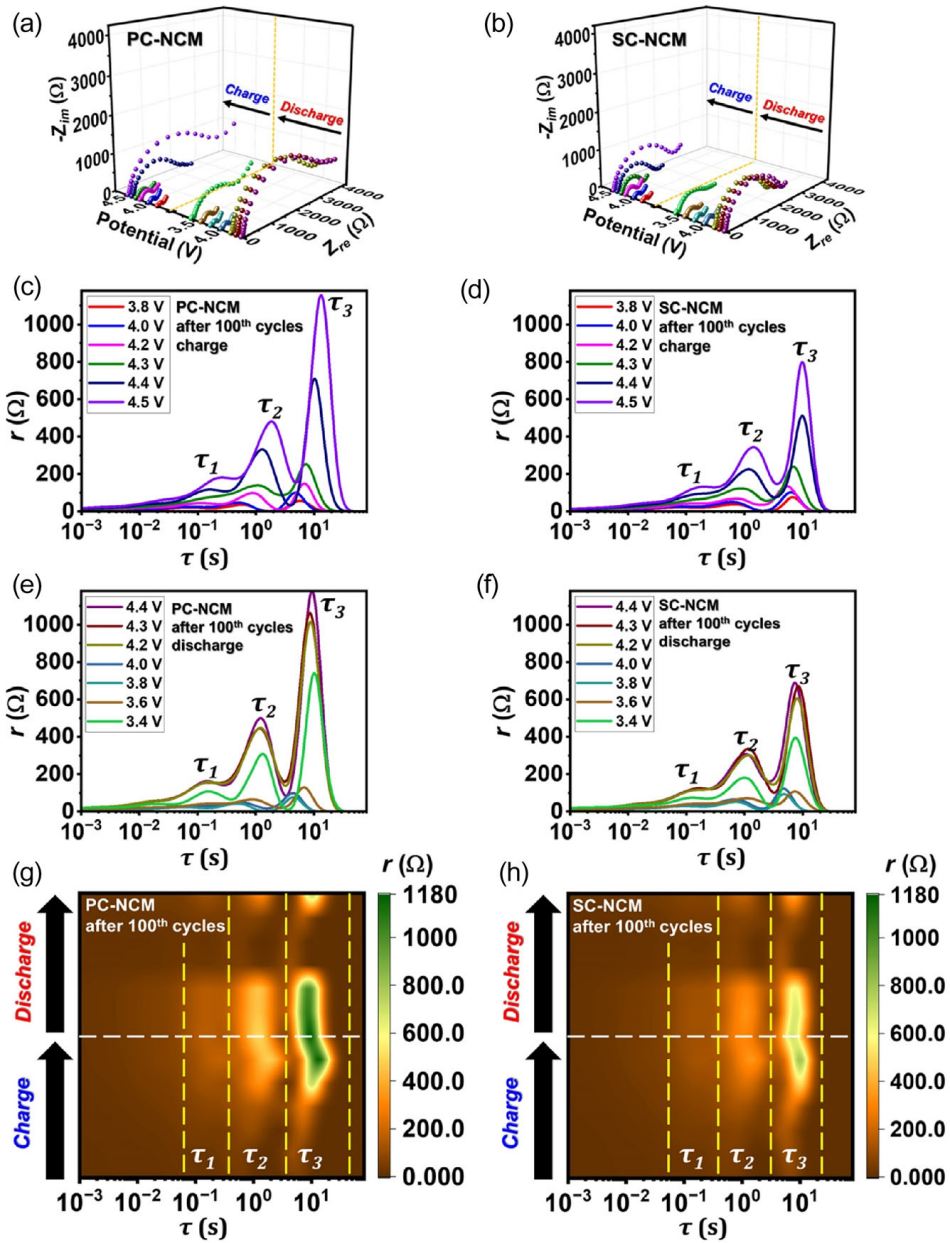
In situ Nyquist plots of (a) PC‐NCM and (b) SC‐NCM obtained at preselected potentials after 100th cycle at 0.1 C. DRT profiles during (c,d) charging and (e,f) discharging. Contour plots of the DRT profiles of (g) PC‐NCM and (h) SC‐NCM.

To demonstrate the origin of structural degradation and the superior cycling stability of SC‐NCM, in situ EIS (electrochemical impedance spectroscopy) and DRT (distribution of relaxation time) analyses at selected potentials after 100 cycles at 0.1 C were conducted for both samples, as shown in Figure 5. In the fresh state after charging to 4.3 V, SC‐NCM exhibited relatively higher resistance than PC‐NCM because of its sluggish bulk Li^+^ diffusion kinetics associated with the single‐crystal structure, as shown in Figure S8. However, during repeated cycling, PC‐NCM became increasingly vulnerable to anisotropic lattice strain accumulated along grain boundaries, and particle cracking subsequently enabled electrolyte penetration into the particles. SEM images after 100 cycles are shown in Figure S9. PC‐NCM underwent severe particle cracking together with cathode electrolyte interphase (CEI) layer formation on the particle surface, as shown in Figure S9a. In contrast, SC‐NCM maintained its particle morphology without noticeable cracking, as shown in Figure S9b. As a result, rapid capacity fading was observed in PC‐NCM during prolonged cycling tests. Nyquist plot variations during the charge and discharge processes are shown in Figure 5a,b. Unlike the initial EIS results after charging, SC‐NCM exhibited an overall lower resistance than PC‐NCM throughout the charge and discharge processes after 100 cycles. To further investigate the complex interfacial evolution during Li intercalation and deintercalation processes, in situ DRT analyses based on EIS spectra were conducted. Distinct relaxation time regions are observed in the DRT results at approximately 10^−1^, 10^0^, and 10^1^ s, labeled as *τ*_1_, *τ*_2_, and *τ*_3_, respectively. According to previous studies, CEI‐layer‐related resistance and charge transfer processes are generally located within *τ* ≈ 10^−2^–10^1^ s, whereas diffusion‐related processes appear at *τ* > 10^1^ s, corresponding to *τ*_1_, *τ*_2_, and *τ*_3_, respectively [56, 57]. As a result of the DRT analysis, *τ*_3_ occupied the largest resistance contribution during cycling. During the charge process, Li^+^ deintercalation induces c‐axis expansion and facilitates Li^+^ diffusion, resulting in relatively low resistance at the initial charge stage. Accordingly, *τ*_1_, *τ*_2_, and *τ*_3_ exhibited relatively low resistance values until charging to 4.3 V. However, excessive Li^+^ extraction and the H2–H3 phase transition at high voltages generated severe lattice strain and structural contraction, thereby increasing the resistance. Unlike *τ*_1_ corresponding to CEI‐layer resistance, which remained relatively stable during charging, *τ*_2_ and *τ*_3_ associated with charge transfer and diffusion processes dramatically increased after charging above 4.3 V and reached maximum values at 4.5 V. Compared with PC‐NCM, SC‐NCM maintained consistently lower resistance throughout the charging process, indicating enhanced tolerance against lattice strain and superior structural stability during repeated cycling. During the discharge process, resistance initially remained relatively high because the contraction of the layered structure hindered Li^+^ intercalation. Therefore, *τ*_1_, *τ*_2_, and *τ*_3_ during the initial discharge stage exhibited relatively high resistance until discharge to 4.2 V, as shown in Figure 5e,f. With continuous lithiation, gradual lattice expansion facilitated Li^+^ diffusion and reduced the resistance. Notably, dramatically increased *τ*_2_ and *τ*_3_ peaks were observed for PC‐NCM and SC‐NCM near 3.4 V. Because Li^+^ sites become increasingly occupied during lithiation near the end of discharge, resistance increased again, implying that Li^+^ intercalation becomes increasingly hindered in PC‐NCM after repeated cycling. For intuitive visualization of resistance evolution during cycling, contour plots of both samples are shown in Figure 5g,h. *τ*_1_ exhibited relatively low resistance, and weak signals were observed in the contour plots. In contrast, distinct *τ*_2_ and *τ*_3_ regions were clearly visualized. Compared with PC‐NCM, SC‐NCM exhibited weaker *τ*_2_ and *τ*_3_ signals with smaller peak shift variations, highlighting its superior structural stability and enhanced tolerance against lattice strain during repeated cycling. Therefore, the in situ DRT results demonstrate that SC‐NCM consistently maintains lower resistance and smaller resistance variation than PC‐NCM, indicating more stable structural properties and reversible Li^+^ transport behavior during prolonged cycling.

**FIGURE 6:**
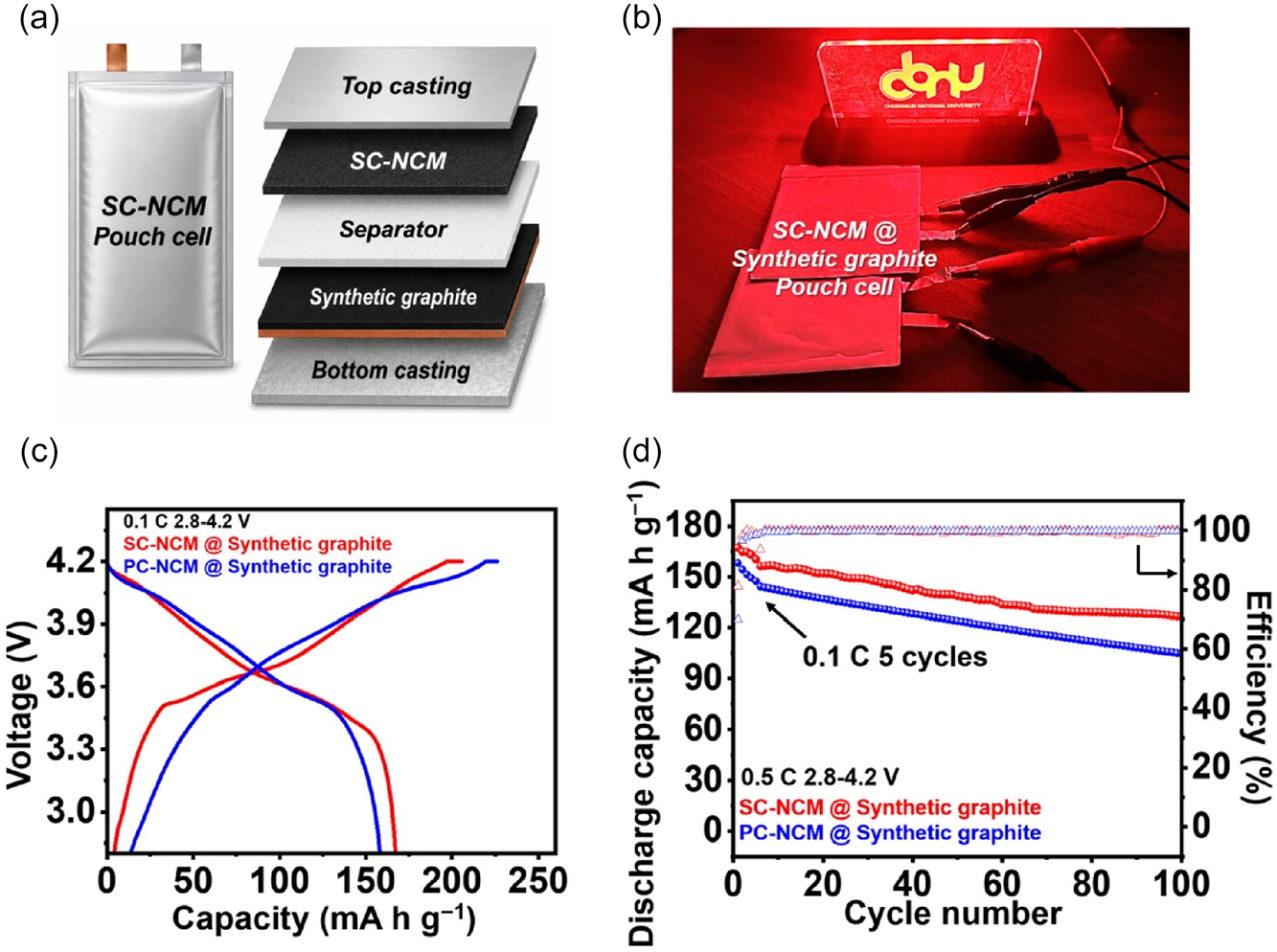
Electrochemical performance of pouch‐type full cell (SC‐NCM/synthetic graphite and PC‐NCM/synthetic graphite): (a) schematic illustration of the pouch‐cell assembly, (b) photograph of an 8 V pouch cell powering an LED lamp, (c) voltage profile during the initial cycles, and (d) cycling performance at 0.5 C.

To demonstrate the practical applicability of SC‐NCM as a cathode material for lithium‐ion batteries, single‐layer pouch‐type full cells were assembled using SC‐NCM and PC‐NCM with synthetic graphite as the cathode and anode materials, respectively, within a voltage window of 2.8–4.2 V. A schematic illustration of the pouch cell assembly is shown in Figure 6a. As a demonstration of practical application, the assembled pouch cell successfully powered an 8 V light‐emitting diode (LED) device, as shown in Figure 6b. The initial charge and discharge curves of SC‐NCM and PC‐NCM pouch cell are shown in Figure 6c. The initial charge and discharge capacities were 205.85/167.18 and 226.30/158.28 mA h g^−1^, corresponding to an ICE of 81% and 70%, respectively. As a result of the cycling test, SC‐NCM and PC‐NCM maintained a capacity retention of 81% and 71%, respectively, until the 100th cycle. These results demonstrate that spray pyrolysis‐based SC‐NCM can serve as a promising cathode material for practical lithium‐ion batteries, while the proposed synthesis strategy can be further extended to various cathode material systems.

## 3 Conclusion

In this study, a direct synthesis strategy for single‐crystalline LiNi_0.8_Co_0.1_Mn_0.1_O_2_ (SC‐NCM) cathode materials was proposed using Li‐containing precursors prepared by spray pyrolysis. Because lithium was homogeneously incorporated within the precursor particles, SC‐NCM was successfully synthesized through direct calcination, with the final crystallization carried out at 900 °C for 3 h and healing process (700 °C for 6 h), without an additional Li‐mixing or washing process. Structural analyses confirmed the formation of single‐crystal particles, whereas PC‐NCM exhibited aggregated polycrystalline grains formed through severe necking during calcination. As a result, SC‐NCM effectively suppressed particle cracking and maintained its structural integrity during repeated cycling. Consequently, SC‐NCM exhibited superior electrochemical performance compared with PC‐NCM. Furthermore, in situ EIS and DRT analyses revealed that SC‐NCM effectively suppressed resistance evolution associated with particle cracking and structural degradation after cycling. These results demonstrate that the Li‐containing precursor strategy provides an effective approach to simultaneously address both the intrinsic limitations of Ni‐rich layered cathode materials and the challenges associated with conventional synthesis processes.

## 4 Experimental Section

### 4.1 Synthesis of Materials

Polycrystalline and single‐crystalline LiNi_0.8_Co_0.1_Mn_0.1_O_2_ cathode materials were synthesized via spray pyrolysis, followed by calcination. A precursor solution was prepared by dissolving stoichiometric amounts (0.5 M) of lithium acetate dihydrate (C_2_H_7_LiO_4_, ≥99.9%, Sigma‐Aldrich), nickel(II) acetate tetrahydrate (Ni(CH_3_COO)_2_·4H_2_O, 97%, Daejung), cobalt(II) acetate tetrahydrate (Co(CH_3_COO)_2_·4H_2_O, 98%, Daejung), and manganese(II) acetate tetrahydrate ((CH_3_COO)_2_·Mn·4H_2_O, 98%, Daejung) in distilled water. To compensate for Li volatilization during calcination, 10% excess Li was added relative to the total transition metal content. During spray pyrolysis, droplets were generated using a 1.7 MHz ultrasonic nebulizer equipped with six vibrators and continuously transported into a quartz tubular reactor maintained at 300 °C (1200 mm in length and 50 mm in inner diameter) using N2 carrier gas at a flow rate of 10 L min^−1^. Rapid solvent evaporation and thermal decomposition of the droplets resulted in the formation of Li‐containing precursor powders. The precursor powders were subsequently calcined in a tubular furnace under an oxygen atmosphere. Based on the TGA results, a multistep heat treatment process was employed before final crystallization, consisting of heating at 200 °C for 1 h, 250 °C for 3 h, 300 °C for 1 h, and 500 °C for 3 h. For the formation of layered cathode materials, they were finally calcined at 780 °C for 15 h to obtain polycrystalline NCM (PC‐NCM), whereas SC‐NCM was synthesized through calcination at 900 °C for 3 h, followed by annealing at 700 °C for 6 h. After calcination, all samples were furnace cooled to room temperature to obtain the final cathode materials.

### 4.2 Characterization

The thermal properties of the synthesized samples were analyzed by TGA (TG 209 F1, NETZSCH) in an air atmosphere over a temperature range from 25 to 800 °C at a heating rate of 5 °C min^−1^. The crystal structures of the samples were examined by XRD using a Rigaku SmartLab diffractometer (9 kW generator, CBO‐3S optics, SC‐70 detector) with Cu K_α_ radiation (*λ* = 1.540593 Å) in the 2*θ* range from 10° to 90°. The morphological characteristics of the samples were investigated via field‐emission scanning electron microscopy (FE‐SEM, S‐4700, Hitachi), and their microstructural features were further analyzed by HR‐TEM (Libra 200 MC, Carl Zeiss) operated at an accelerating voltage of 200 kV. Transmission electron microscopy (TEM) specimens were prepared using a FIB system (Helios 5 UX, Thermo Fisher Scientific) installed at the Korea Basic Science Institute (KBSI, Daejeon; Equipment code: EM01). To improve lattice visibility while preserving structural fidelity, only mild FFT‐based noise filtering and linear brightness adjustments were applied using a Gatan Digital Micrograph. Elemental distributions of the SC‐NCM and PC‐NCM samples were examined by TEM‐EDS using a JEM‐ARM200F (NEOARM, JEOL). SEM, EDS, cross‐sectional, and 3D structural analysis were performed using a cryogenic plasma FIB (Helios 5 Hydra CX, NFEC‐2025‐12‐311498) at Advanced Energy Research Institute (AERI), Chungbuk National University. XPS of the samples was performed using ESCALAB‐250 with Al K_α_ radiation (1486.6 eV). FE‐SEM (FEI Quattro S) equipped with EBSD (EDAX Velocity Super) was operated at 10 kV to observe SC‐NCM and PC‐NCM particles/grains.

### 4.3 Electrochemical Measurements

All electrochemical properties were investigated using coin‐type cells (CR2032), which were assembled in an argon‐filled glove box to ensure an inert environment. All electrodes were punched to a diameter of 14 mm. To prepare the cathode electrodes, cathode powder, Super P, and polyvinylidene fluoride (PVDF, Kureha KF9700) were mixed in a weight ratio of 9:0.5:0.5. The mixture was then dispersed in N‐methyl‐2‐pyrrolidone (NMP) solvent to form a homogeneous slurry. The slurry was coated onto aluminum foil and dried at 60 °C for 12 h. For half‐cell tests, lithium metal and a polypropylene separator were used as the counter electrode and separator, respectively. The electrolyte for half‐cell measurements consisted of 1.2 M LiPF6 dissolved in an ethylene carbonate/ethyl methyl carbonate (EC/EMC, 3:7 v/v) mixture with 2 wt.% vinylene carbonate (VC) as an additive. For pouch‐type full‐cell evaluation, the synthesized cathode was paired with a synthetic graphite anode. The anode slurry was prepared by mixing graphite powder (90 wt%), Super P (4 wt%), and PVDF (6 wt%) in NMP, followed by coating onto copper foil and drying under vacuum at 60 °C for 10 h. The cathode electrode was fabricated using the same procedure described above, with an electrode area of 16.5 cm^2^ and an active material loading of approximately 4.5 mg cm^−2^. The NP ratio was carefully controlled at 1.10. The same electrolyte as that used in the half‐cells was employed, with a total volume of 160 μL. The electrochemical performance of the cells was evaluated after resting for at least 24 h at room temperature. Galvanostatic charge–discharge tests were conducted within a voltage range from 2.7 to 4.3 V for half‐cells and 2.8–4.2 V for full cells, with an initial current density of 0.1 C (1 C = 200 mA g^−1^). CV measurements were performed at a scan rate of 0.1 mV s^−1^. In situ EIS (ZIVE SP1) analysis was performed at preselected potentials during cycling at a current density of 0.1 C. The EIS measurements of the electrode were performed over a frequency range from 0.001 Hz to 100 kHz. The DRT transformation was performed using MATLAB software, with all parameters kept consistent throughout the calculations to ensure the reliability of the DRT results.

## Funding

This study was supported by the National Research Foundation of Korea (RS‐2023‐00217581), Ministry of Science and ICT, South Korea (RS‐2023‐00304768), and Ministry of Education of South Korea (RS‐2025‐02317677, 2026‐RISE‐11‐014‐06).

## Conflicts of Interest

The authors declare no conflicts of interest.

## Supporting information

Supplementary Material

## Data Availability

The data that support the findings of this study are available from the corresponding author upon reasonable request.
