# Supplementary material for: Direct Synthesis of Single‐Crystalline LiNi0.8Co0.1Mn0.1O2 Cathodes From Lithium‐Containing Precursors via Spray Pyrolysis
Source: Small Sci. 2026 Sep 3;6(9):e70397. doi: 10.1002/smsc.70397 (PMC13574055; doi:10.1002/smsc.70397)
Supplement: Supplementary file 1 [file SMSC-6-e70397-s001.pdf]

((Supporting Information can be included here using this template))

Copyright WILEY-VCH Verlag GmbH & Co. KGaA, 69469 Weinheim, Germany, 2013.

Supporting Information

Direct Synthesis of Single-Crystalline $LiNi_{0.8}Co_{0.1}Mn_{0.1}O_2$ Cathodes from Lithium-Containing Precursors via Spray Pyrolysis

Tae Ha Kim, Yeon Oh Lee, Yeong Beom Kim, Hyungsub Kim, Jung Sang Cho, Sang Mun Jeong*, Yun Chan Kang*, and Gi Dae Park**

T.H. Kim, Prof. G.D. Park*
Department of Urban, Energy, and Environmental Engineering,
Chungbuk National University,
Chungdae-ro 1, Seowon-gu, Cheongju,
Chungbuk 28644, Republic of Korea
E-mail: gdpark@chungbuk.ac.kr

Y.O. Lee, Prof. G.D. Park*
Department of Advanced Materials Engineering,
Chungbuk National University,
Chungdae-ro 1, Seowon-gu, Cheongju,
Chungbuk 28644, Republic of Korea

Y.B. Kim, Prof. Y.C. Kang*
Department of Materials Science and Engineering,
Korea University,
Anam-Dong, Seongbuk-Gu,
Seoul 136-713, Republic of Korea.
E-mail: yckang@korea.ac.kr

H.S. Kim
Neutron Science Division, Korea Atomic Energy Research Institute (KAERI),
111 Daedeok-daero 989, Beon-Gil, Yuseong-gu,
Daejeon 34057, Republic of Korea,

Prof. J.S. Cho*
Department of Engineering Chemistry,
Chungbuk National University,
Chungdae-ro 1, Seowon-Gu, Cheongju,
Chungbuk 28644, Republic of Korea.
E-mail: jscho@cbnu.ac.kr

Prof. S.M. Jeong*
Department of Chemical Engineering,

Chungbuk National University,
Chungdae-ro 1, Seowon-gu, Cheongju,
Chungbuk 28644, Republic of Korea
E-mail: smjeong@chungbuk.ac.kr

H.S. Kim, Prof. J.S. Cho*, Prof. S.M. Jeong*, and Prof. G.D. Park*,
Advanced Energy Research Institute, Chungbuk National University,
Chungdae-ro 1, Seowon-gu, Cheongju,
Chungbuk 28644, Republic of Korea

Keywords: Spray pyrolysis, Li-containing precursor, Ni-rich layered cathode, Single-crystalline cathode, Li-ion batteries.

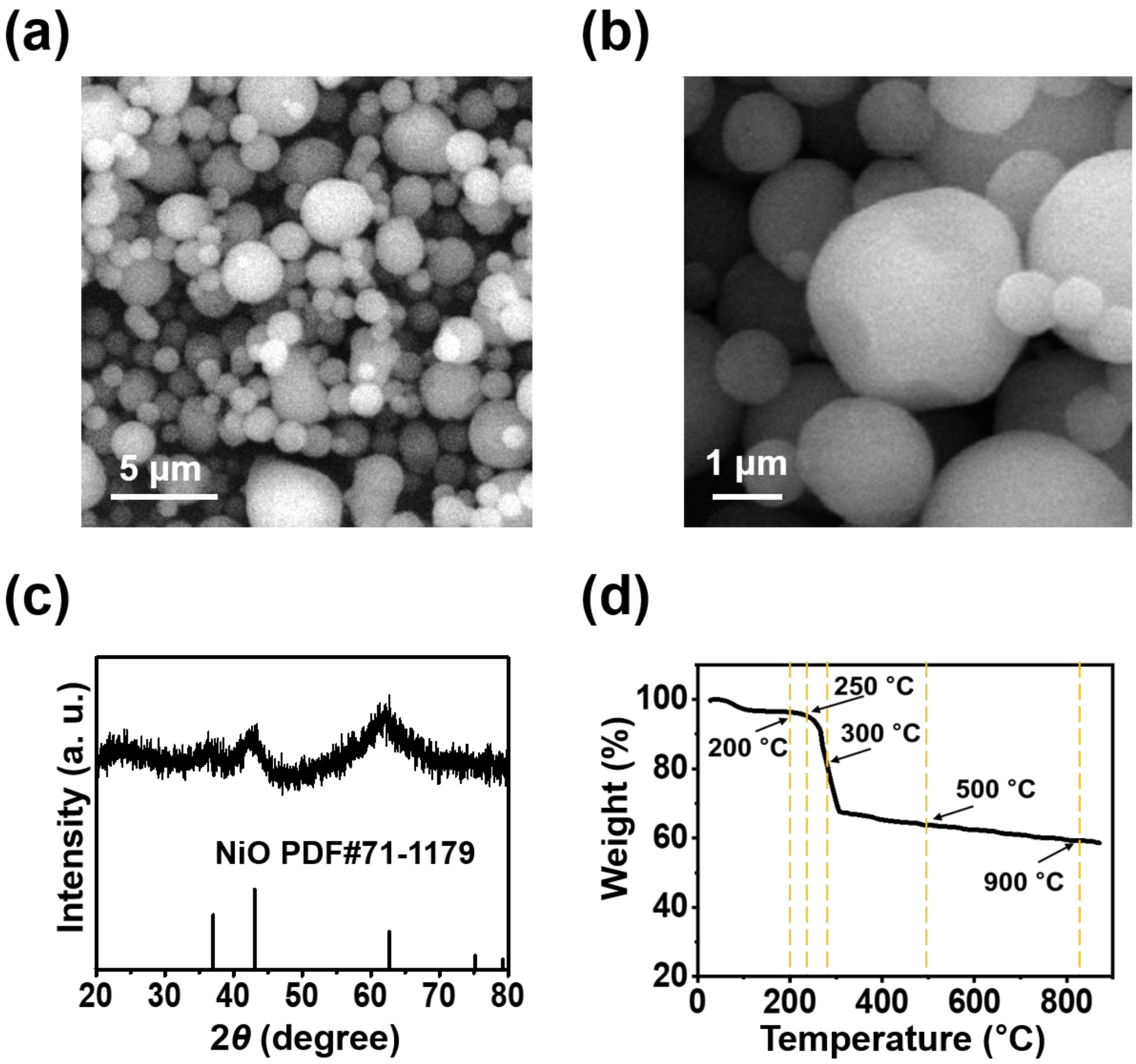

Figure S1. a, b) SEM images, c) XRD patterns, d) TGA curves of NCM precursor.

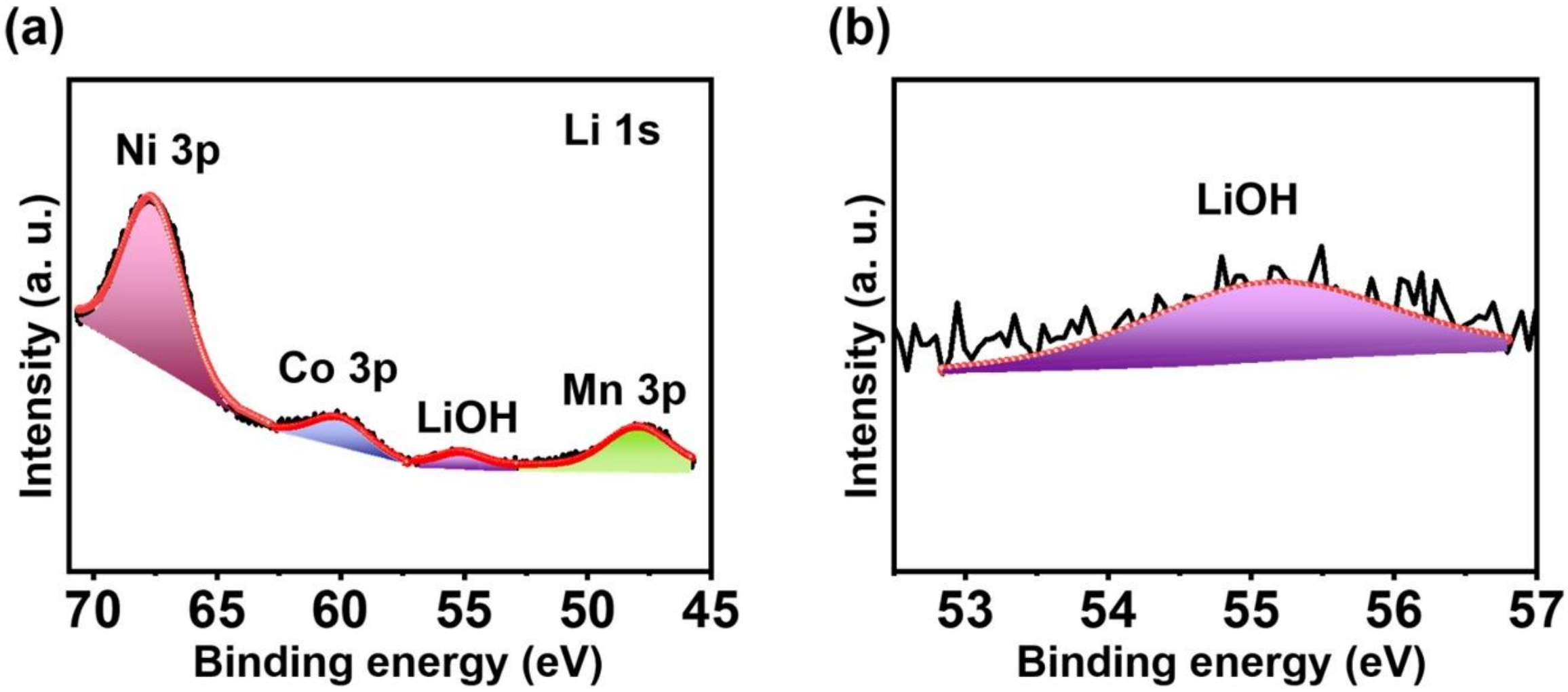

Figure S2. XPS analysis of NCM precursor (a-b) Li 1s spectra.

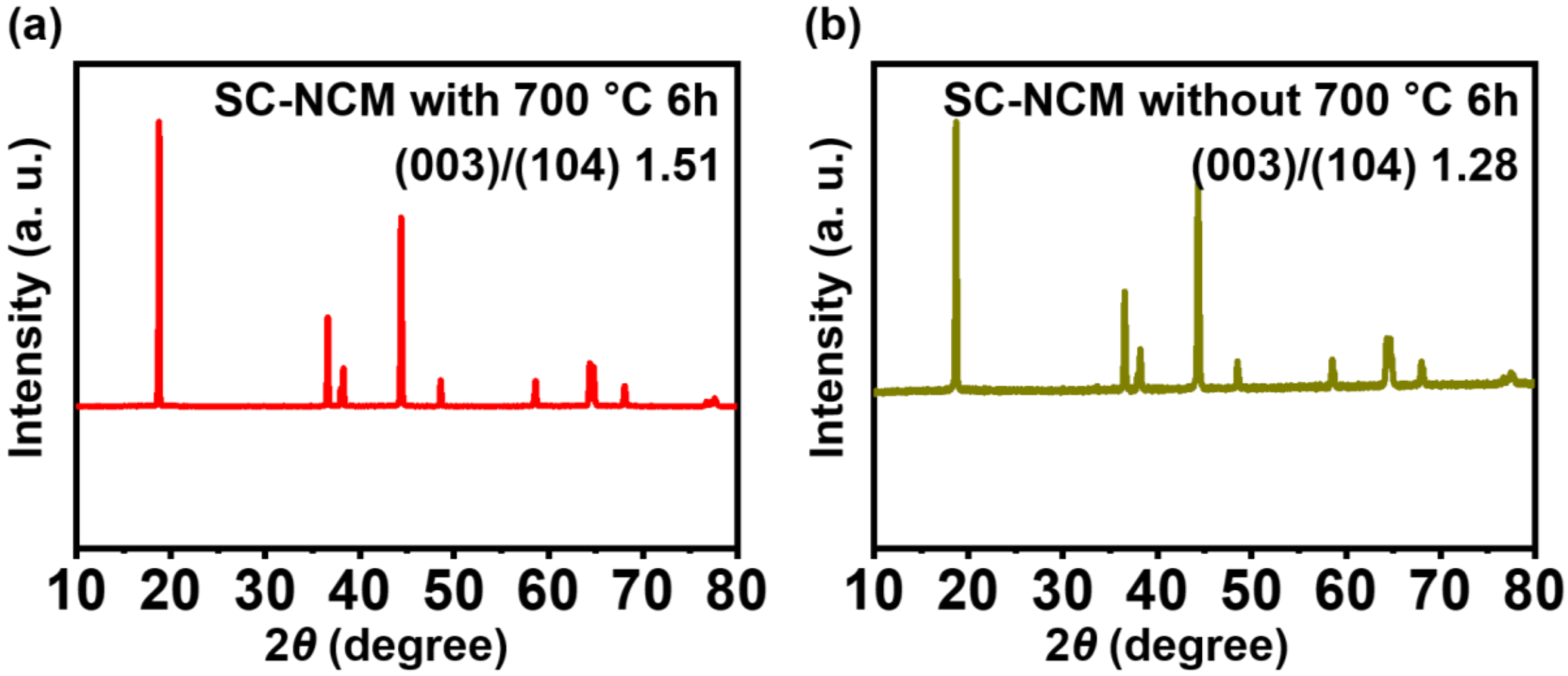

Figure S3. XRD patterns of a) SC-NCM compared with b) SC-NCM without 700 °C 6h.

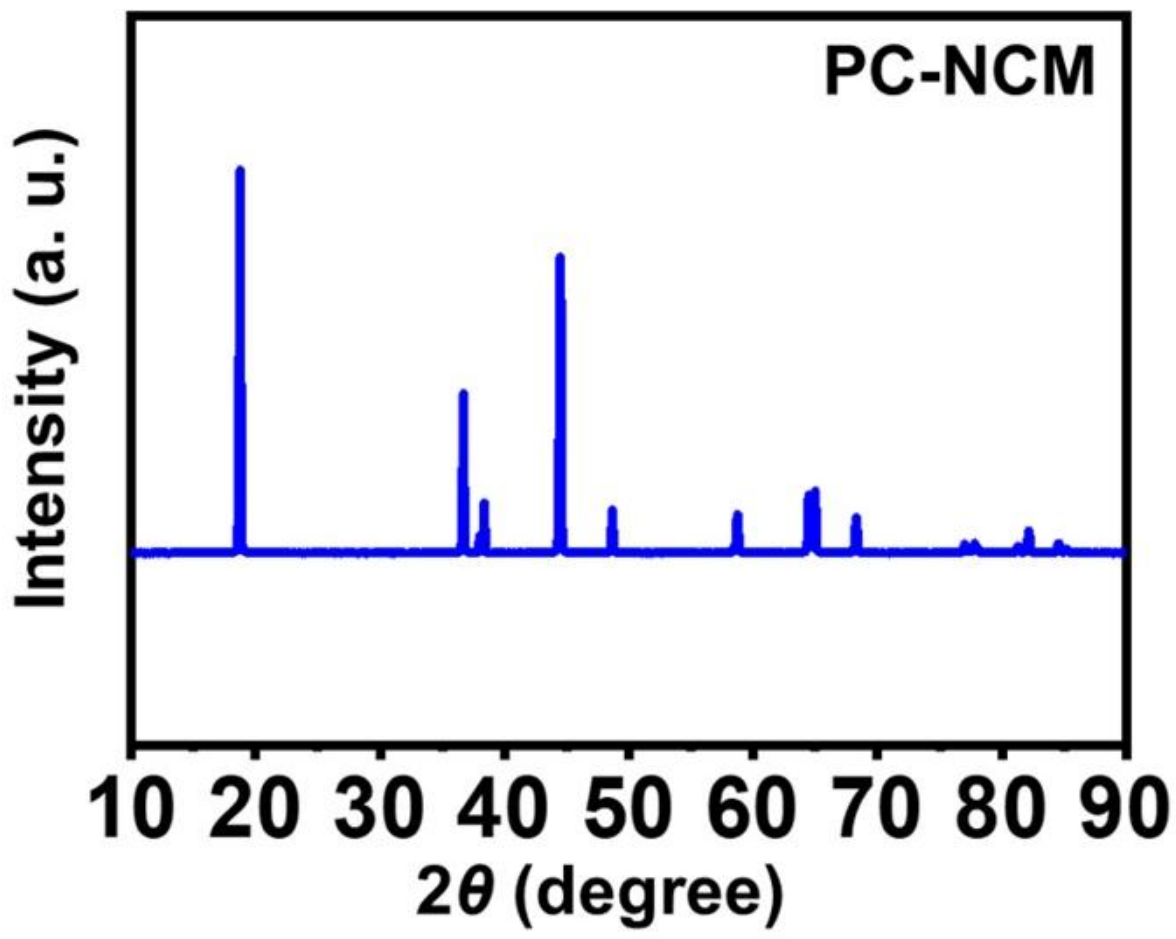

Figure S4. XRD patterns of PC-NCM.

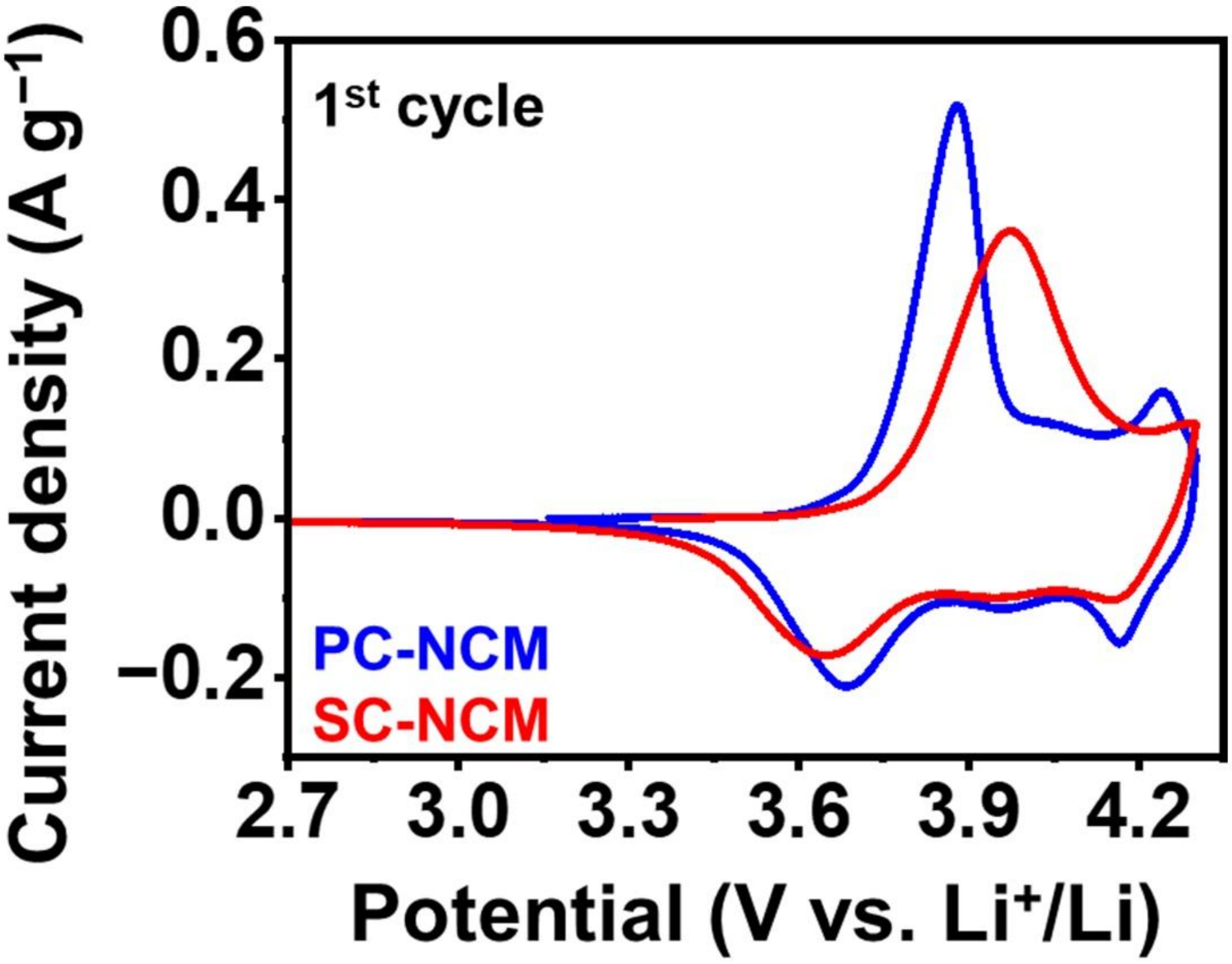

Figure S5. CV curves of PC-NCM and SC-NCM within 2.7–4.3 V at a scan rate of 0.1 mV s^{-1}.

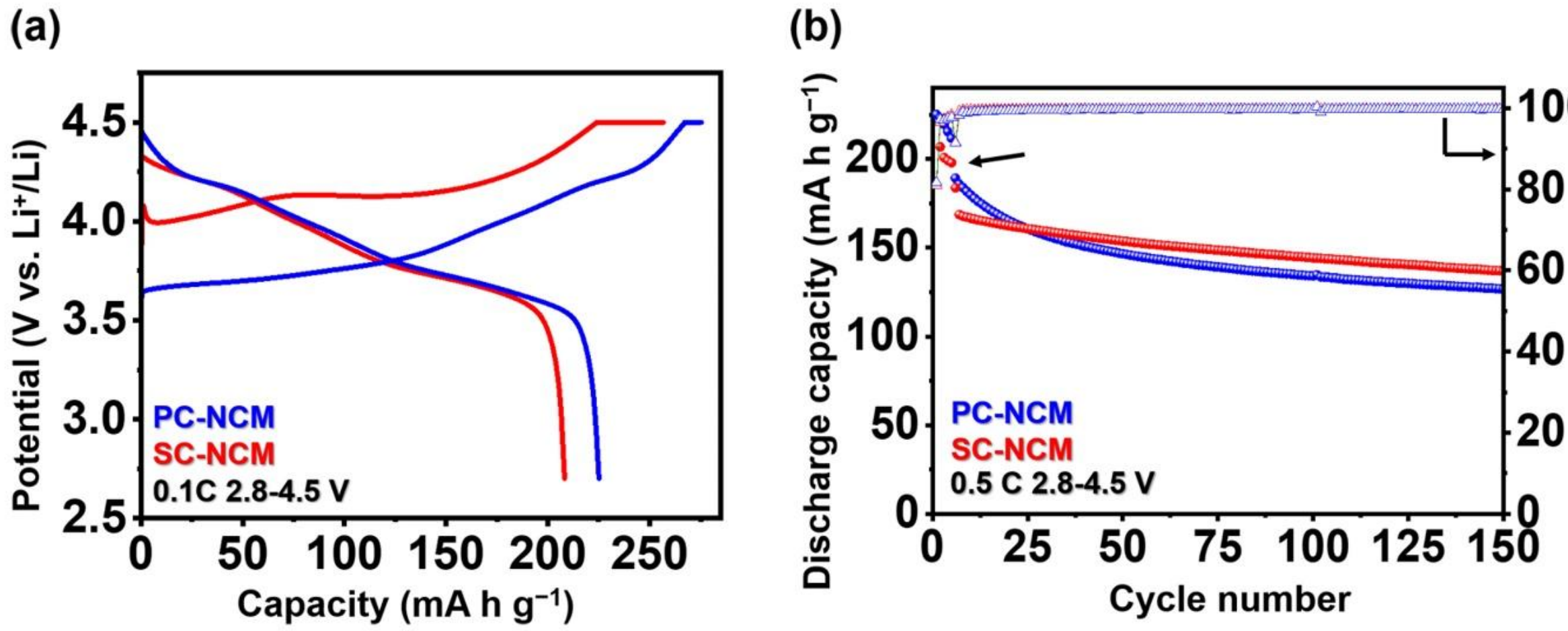

Figure S6. a) voltage profile during the initial cycles and b) cycling performance of PC-NCM and SC-NCM measured with 4.5 V cutoff voltage at 0.5 C.

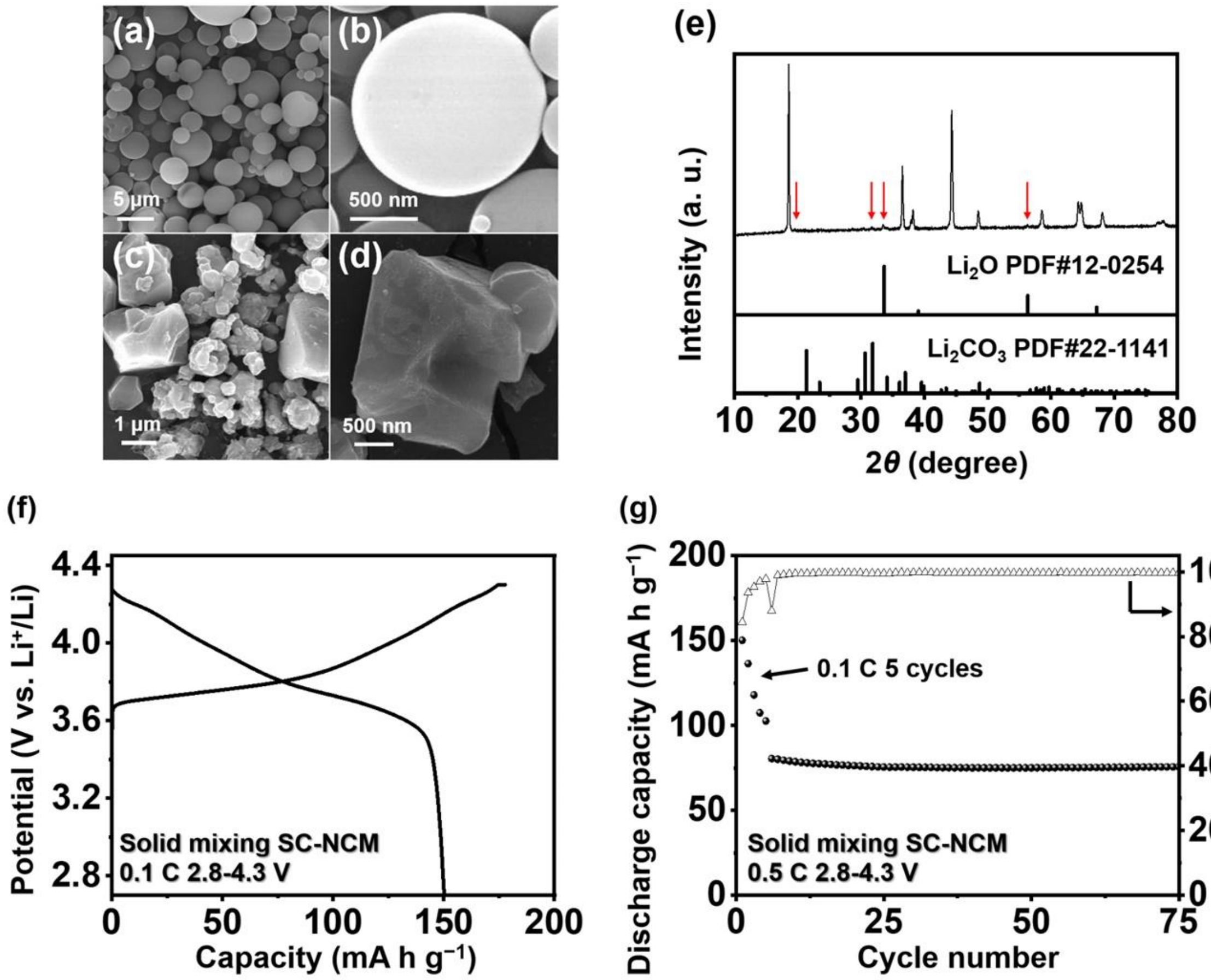

Figure S7. SEM images of a, b) precursor without Li, c, d) solid mixing SC-NCM. e) XRD patterns of solid mixing SC-NCM. f) Voltage profile during the initial cycles and g) cycling performance of solid mixing SC-NCM measured with 4.3 V cutoff voltage at 0.5 C.

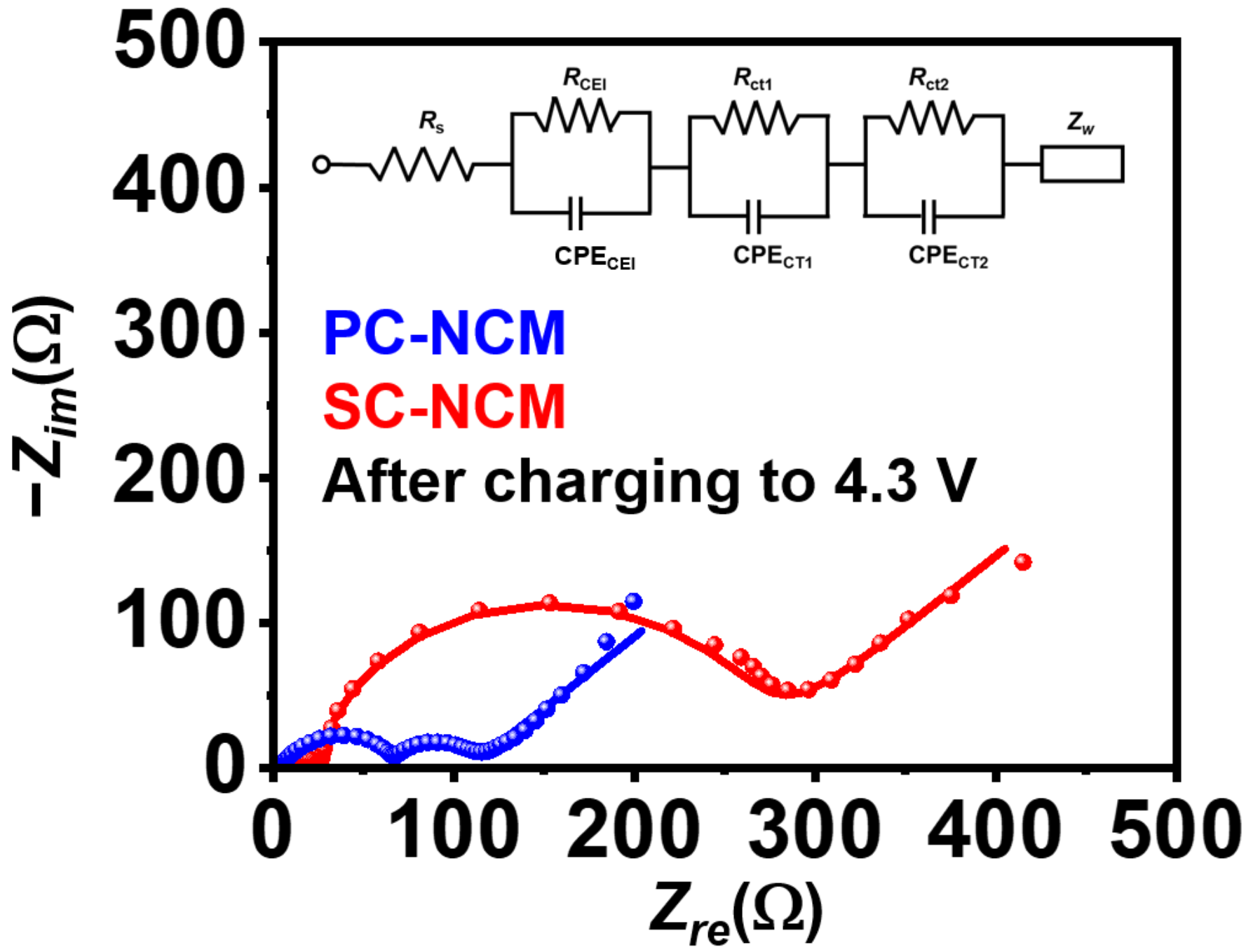

Figure S8. Nyquist plots (measured points and fitted lines) of PC-NCM and SC-NCM after charge at 4.3 V (inset Equivalent circuit cell diagram).

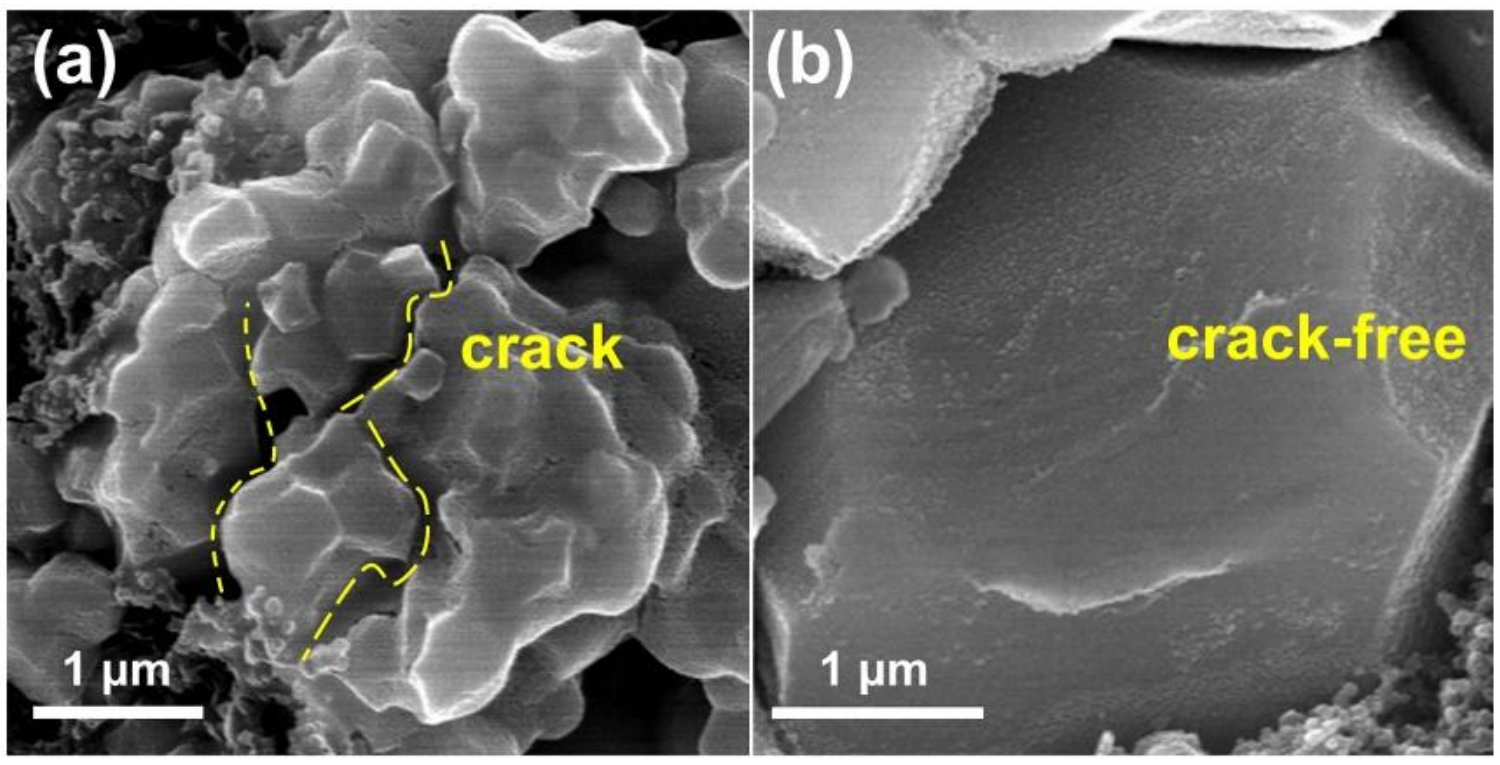

Figure S9. SEM images of a) PC-NCM and b) SC-NCM after 100 cycles at 0.1 C.

Table S1 ICP data of NCM precursor, PC-NCM, and SC-NCM.

ICP data	NCM precursor	PC-NCM	SC-NCM
**Li**	1.12	1.020	1.006
**Ni**	80.803	79.728	79.034
**Co**	10.062	10.216	10.208
**Mn**	9.135	10.056	10.757

Table S2 Rietveld refinement results of PC-NCM and SC-NCM.

**R-3m**	**PC-NCM**	**SC-NCM**
**a, b (Å)**	2.86977 (3)	2.87986 (3)
**c (Å)**	14.1949 (2)	14.2198 (3)
**Vol. ($Å^3$)**	101.241 (2)	102.133 (2)
**Cation mixing**	1.0%	2.8%
**R_p (%)**	2.50	3.12
**R_{wp} (%)**	4.56	5.68
**R_F (%)**	1.49	2.50
